# Projected speaker numbers and dormancy risks of Canada’s Indigenous languages

**DOI:** 10.1098/rsos.241091

**Published:** 2025-02-19

**Authors:** Michaël Boissonneault, Adam Tallman, Volker Gast, Simon J. Greenhill

**Affiliations:** ^1^ Department of demography and population sciences, Université de Montréal, Montreal, Canada; ^2^ Department of Linguistics and Philology, Uppsala Universitet, Uppsala, Sweden; ^3^ Department of English and American Studies, Friedrich-Schiller-Universität Jena, Jena, Germany; ^4^ School of Biological Sciences, The University of Auckland, Auckland, New Zealand; ^5^ Department of Linguistic and Cultural Evolution, Max Planck Institute for Evolutionary Anthropology, Leipzig, Germany

**Keywords:** international decade of indigenous languages, linguistic diversity, population projection, census data, language endangerment, indigenous languages of Canada

## Abstract

UNESCO launched the International Decade of Indigenous Languages in 2022 to draw attention to the impending loss of nearly half of the world’s linguistic diversity. However, how the speaker numbers and dormancy risks of these languages will evolve remains largely unexplored. Here, we use Canadian census data and probabilistic population projection to estimate changes in speaker numbers and dormancy risks of 27 Indigenous languages. Our model suggests that speaker numbers could, over the period 2001–2101, decline by more than 90% in 16 languages and that dormancy risks could surpass 50% among five. Since the declines are greater among already less commonly spoken languages, just nine languages could account for more than 99% of all Canadian Indigenous language speakers in 2101. Finally, dormancy risks tend to be higher among isolates and within specific language families, providing additional evidence about the uneven nature of language endangerment worldwide. Our approach further illustrates the magnitude of the crisis in linguistic diversity and suggests that demographic projection could be a useful tool in assessing the vitality of the world’s languages.

## 1. Background

The approximately 7000 languages currently spoken worldwide represent a diversity that is important for speech communities, because of the cultural value they bear and the source of health and wellbeing they furnish [1–4]. This diversity is also important for science, as researchers from diverse fields rely on it to better understand human cognition, history and the natural world [5–9]. This diversity is under threat, however, as more than 40% of the world’s languages are considered endangered [10]. Recognizing the importance of linguistic diversity for communities and scientific understanding, in 2022, UNESCO launched the International Decade of Indigenous Languages to draw attention to ‘the critical loss of Indigenous languages and the urgent need to preserve, revitalize and promote’ them [11].

Language speaker numbers worldwide are regularly updated in Ethnologue, one of the most comprehensive inventories of the world’s languages [12], and were supplied by the Endangered Languages Project and UNESCO’s World Atlas of Languages [13,14]. These inventories assign languages to endangerment categories that account for their level of intergenerational transmission and domains of use [14–16]. However, these assessments only provide a snapshot of a language’s situation in the recent past and do not necessarily provide clear information about how it may evolve in the future.

Two studies have provided global estimates of the future of linguistic diversity, basing their calculations on Ethnologue’s classification by endangerment category and stylized facts about the age of the current speakers and their expected longevity [17,18]. In both cases, the results have suggested that approximately 20% of the languages currently spoken could disappear before the end of the current century—essentially the proportion of languages that, according to Ethnologue, are at present no longer being learned by children. Mathematical models resting on differential [19–24] or agent-based simulations [25–28] have also been developed to study the mechanisms through which languages come to compete for speakers in a given area. However, these models are either purely theoretical or fitted to data collected among a handful of better-documented languages [29].

Here, we use a demographic projection model [30] to study the future of 27 Indigenous languages of Canada. Similar to agent-based models, our model focuses on speakers as the unit of analysis. Unlike these models, however, our model does not establish any rules about how speakers interact with each other and focuses instead on their longevity and propensity to have children to whom they transmit their language. Our model concentrates on a person’s first language and how it is transmitted across generations. Key parameters include mortality, which determine a speaker’s survival over time, and language-specific mean numbers of child speakers per adult speaker, which reflects the number of children that each speaker will have in their life course to whom they will transmit their language. These are in turn determined using language-specific probabilistic baseline populations, which we estimated from census data collected at five points in time using an approach that we developed for this study. In this model, a language’s prospects are thus determined entirely empirically from observed past mortality, fertility and language transmission behaviours.

Our model thus considers the same mechanisms of language endangerment as the two previous studies that have provided global estimates of the future of linguistic diversity (i.e. mortality and intergenerational transmission), but provides more precise insights into a language’s prospects as it is not limited by discrete endangerment categories. Another of our model’s advantages lies in its use of bespoke mortality information instead of stylized facts, leading to more accurate estimates of how long speakers might still live. Additionally, our model provides as output projected numbers of speakers by age in the subsequent period of five years together with projection intervals. This represents arguably more practical information than the one provided by mathematical models, which typically consist of prognoses of whether two languages can coexist in the same area and under which circumstances.

We estimated baseline populations and rates of intergenerational transmission of language-specific counts of speakers by groups of five years of age collected during the Canadian censuses of 2001, 2006, 2011, 2016 and 2021 [31]. This type of data contains more precise information on the age of speakers than classifications by endangerment categories and has the advantage of having been collected simultaneously among the country’s entire population using standardized questionnaires. Also, although caution is often advocated when using census data on language usage [32], by international standard, data collected by the Canadian census bolster comparatively high response rates and benefit from extensive quality checks [33,34].

Nonetheless, census data always suffer from a certain degree of error and can obviously not capture the same subtleties that are typically captured by ethnographic studies. To maximize our use of the Canadian census data and minimize the impact of potential errors on our results, we first carefully examined the consistency of the data over time and set aside cases where the data presented implausible patterns. We then developed a statistical model that allowed us to estimate for each language probabilistic baseline populations that captured the uncertainty affecting the data. This uncertainty was then transparently propagated through all stages of the analysis, from the estimation of the mean number of child speakers per adult speaker to the projection of the populations by five-year age groups. The use of a Monte Carlo method further allowed us to consider the impact of chance on a language’s prospects and to estimate its dormancy risk [35].

Human settlement of the current Canadian territory began alongside the colonization of the rest of the Americas as early as 30 000 years ago [36]. The establishment of the first European settlers in the 17th century and the ensuing forced displacements, wars and epidemics decimated Indigenous populations and linguistic diversity, with estimates suggesting a decrease of up to 90% in population sizes [37–39]. The residential school system that prevailed for most of the 19th and 20th centuries forcibly disconnected thousands of Indigenous children from their language and culture, negatively impacting the intergenerational transmission of most languages. The Truth and Reconciliation Commission of Canada proposed in its 2015 report 94 calls to action ‘to further reconciliation between Canadians and Indigenous Peoples’ [40]—including funds for language revitalization—but progress has been uneven [41]. Despite this adversity, more than 60 Indigenous languages continue to be spoken across the country and although recent trends indicate a decline in the total number of people reporting an Indigenous language as their first language, experiences vary greatly across languages [42].

In this study, a speaker refers to a person who learned a given language in childhood and still understands it at the moment of the census. Following current practice, we use the term ‘dormant’ (rather than dead or extinct) for languages without living speakers [43].

## 2. Material and methods

We developed an individual-based, cohort-component demographic projection model with the aim of projecting, for each language separately, speaker numbers in groups of five years of age, at intervals of five years between 2001 and 2101. Components included the birth of new speakers and their death, with the birth of speakers being primarily determined by the trends in the mean number of child speakers per adult speaker that we derived from the data specific to each language. Our model assumes that languages are only transmitted from parents to their children and that children who acquire a language in childhood retain its use throughout their life.

**Figure 1.**
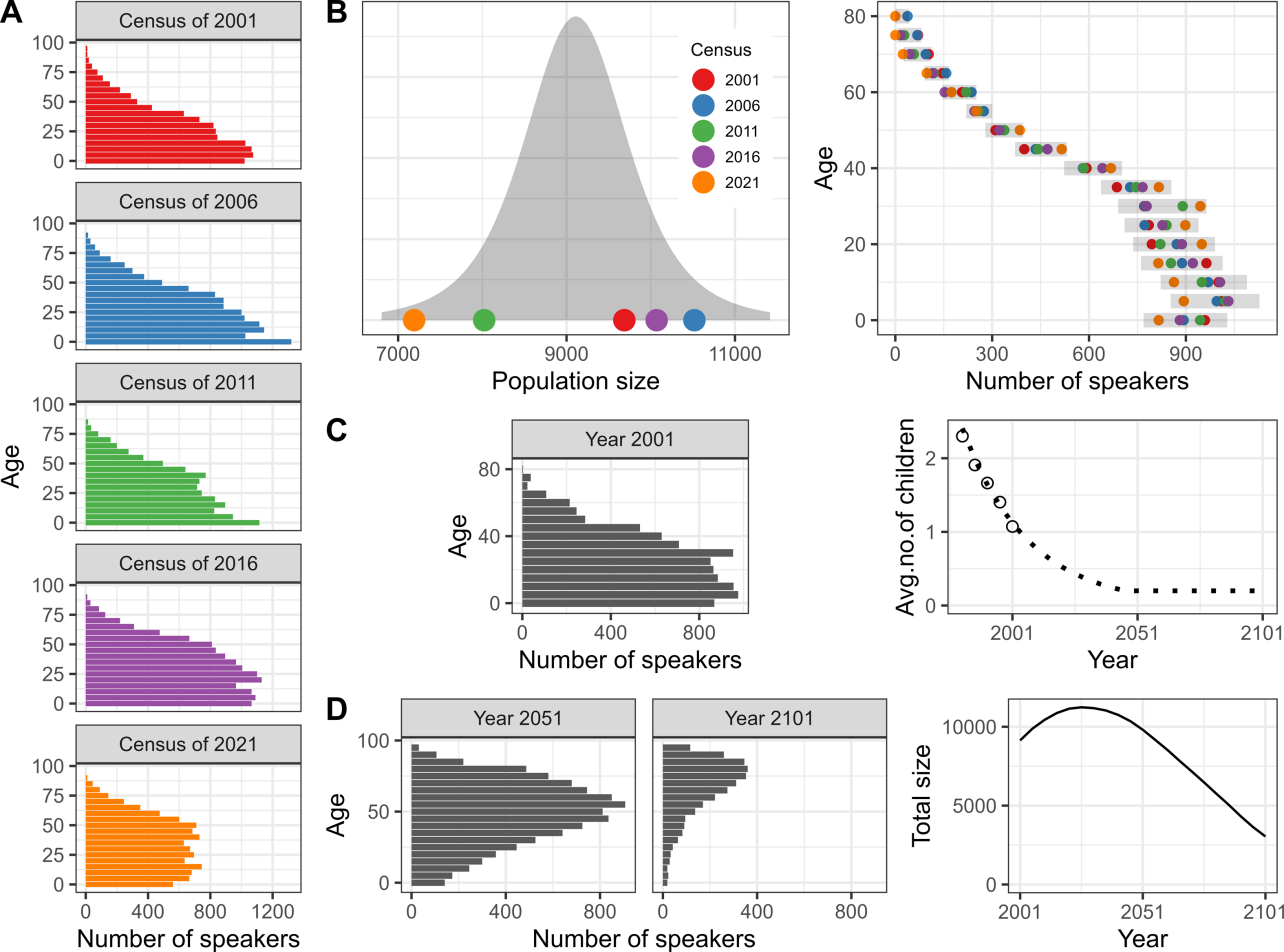
Steps leading to the estimation of the number of speakers for the Oji-Cree language. (A) shows the number of speakers observed during the censuses of 2001, 2006, 2011, 2016 and 2021 by groups of five years of age for the Oji-Cree language. (B) shows the estimation of the baseline population in the year 2001 at the hand of a t-distribution for the total size (left) and a series of negative binomial distributions (one for each five-year age group) (right). The shaded area on the left represents the density (t-distribution) while the shaded bands on the right show the values between the 10th and 90th percentiles (negative binomial distributions). The points represent the values observed at each census recast in the year 2001. (C) shows on the left the baseline population resulting from a specific series of random draws taken from each age-specific negative binomial distribution combined with a specific single random draw from the t-distribution. The former determined the speakers’ distribution across age groups, the latter the total population size in the year 2001. From the resulting population by age, estimates of the mean number of child speakers per adult speaker were obtained for the years 1981, 1986, 1991 and 1996 (empty circles) to which an exponential model was fitted and extrapolated (dotted line) (Panel C, right). (D) shows the population numbers resulting from the application of the projection model to the baseline population in C with the resulting counts by five-year age groups in the years 2051 and 2101 (left) and the total population in each projection year (right). Steps C and D were repeated 3000 times; results for all languages are shown in figure 3 and table 1.

We first defined probabilistic baseline populations in the year 2001 for each language. These were obtained from data collected during the Canadian censuses of 2001, 2006, 2011, 2016 and 2021. Based on these, we used indirect estimation techniques to estimate past trends in the mean number of child speakers per adult speaker for each language and mathematical modelling to extrapolate these into the future. The values thereby obtained were then combined with standard age- and period-specific fertility schedules, and births were assigned to speakers in a cohort-component projection model. The same speakers were then subject to death following standard age- and period-specific schedules that were selected based on the most recent information on the mortality of the Indigenous peoples of Canada. Finally, we took an individual-based approach to estimate the occurrence of births and deaths within the cohort-component model, to account for the stochasticity affecting small populations and assess the risk of languages to become dormant in the future. Methods are summarized graphically in figure 1 using the population of Oji-Cree speakers as an example and schematically in electronic supplementary material, figure S1.

Below we describe in more detail (i) the census data on the first language, (ii) the estimation of the baseline populations, (iii) the estimation of the trends in the mean number of child speakers per adult speaker, (iv) the recast procedure used to obtain both the baseline populations and the trends in the mean number of child speakers per adult speaker , (v) the projection model, and (vi) the analyses.

### 2.1. Data

We obtained from Statistics Canada counts of speakers by Indigenous first language for the censuses of 2001, 2006, 2011, 2016 and 2021, for each five-year age group included between 0 and 4 and the open-ended category ‘100 years or older’ [44]. Counts are based on answers to the question ‘What is the first language that this person first learned at home in childhood and still understands?’ included in the census’ so called long-form questionnaire [34]. This questionnaire is distributed to one household out of five at the national level but to each household located on an Indian reserve or in remote areas, including in Nunangat (i.e. the Canadian polar region).

Answers to the question on the first language may include English, French or Other; respondents who select 'Other’ are asked to indicate their language in print letters. Following each census, counts are published according to a list of languages maintained by Statistics Canada. The number of languages appearing on this list increased from 35 to 74 between 2001 and 2021, an increase that is mainly due to the use of more specific language denominations over time. For example, whereas counts were published jointly for the languages Innu and Naskapi following the censuses of 2001 and 2006, they were published separately following the censuses conducted from 2011 onward.

**Figure 2.**
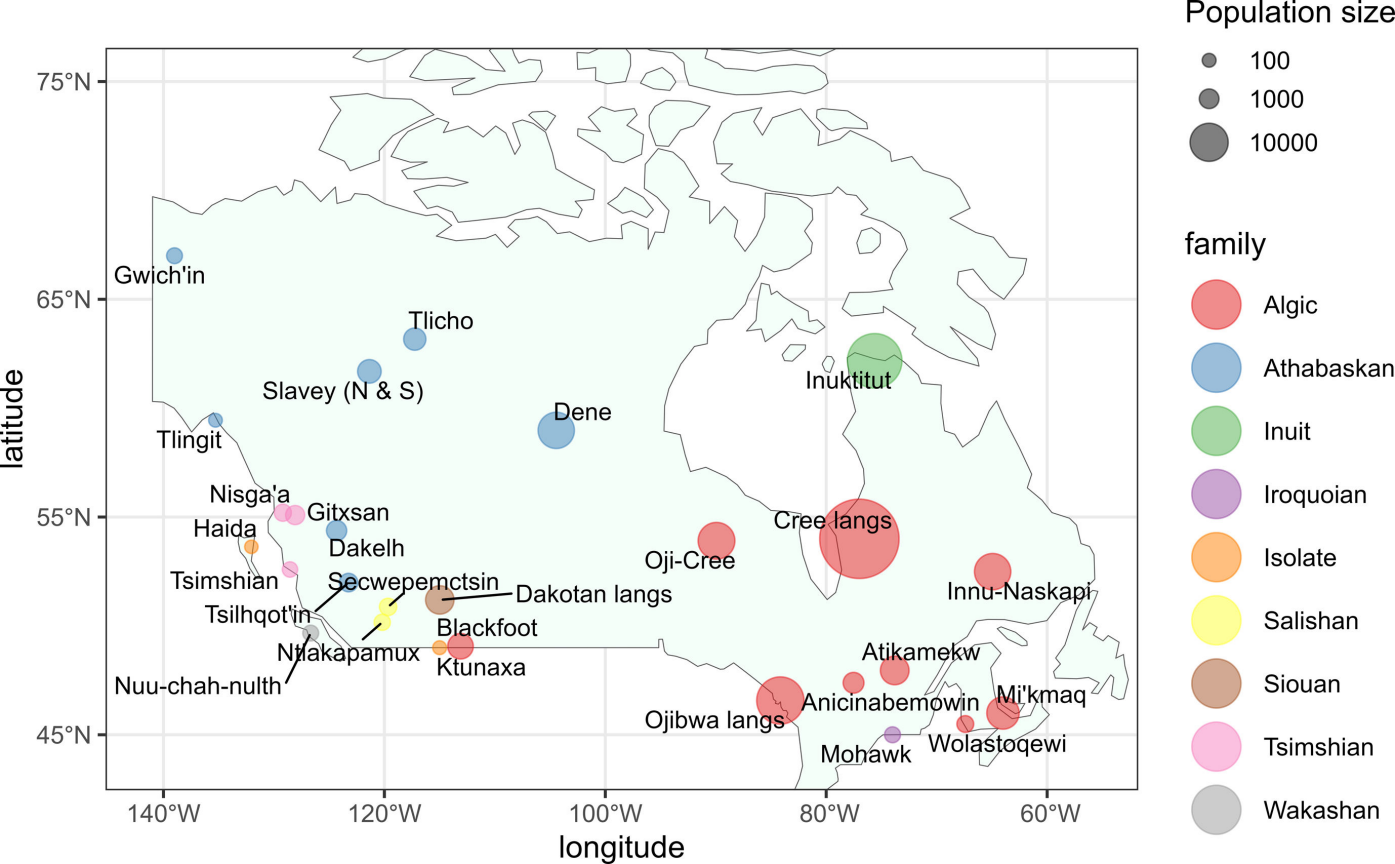
Geographic distribution and population sizes of the 27 languages included in the analysis. The size of the dots represents the size of the baseline population in 2001, their colour the linguistic family. Locations were obtained from Ethnologue with modifications by the authors, the families from [34]. Locations do not necessarily represent the speakers’ location during censuses.

We consider in this study 27 languages (or groups of languages) for which we were able to identify counts referring to consistent categories between 2001 and 2021 (figure 2 and electronic supplementary material, table S1). These cover about half of the Canadian Indigenous languages identified in the ISO 639-3 standard. These counts refer either to the speakers of what are typically considered as specific languages (such as Atikamekw or Mi’kmaq), or to the speakers of what are typically considered as groups of languages (such as the Cree languages).^1^ We used in this study counts referring to respondents who provided a single response to the question of the first language.

All speaker counts refer to the population residing in Canada on the 1st of July of each census year. Following each census, counts are subject to various quality checks by Statistics Canada. Weighting and imputation are used to correct for sampling and non-response, where applicable. Counts are additionally subject to random rounding to the nearest unit of five in order to protect the respondents’ privacy [31].

### 2.2. Baseline populations

We used the census information to estimate baseline populations in the year 2001 separately for each language. The census data allows us to observe the same population (defined by the language spoken by its members) at five points in time. Once we have identified a speaker’s birth cohort and corrected for the mortality that affects the counts, these five observations should provide similar estimates for the population of a given language. However, imperfect census coverage and reporting problems translate into some variation; these errors can become particularly salient when we are dealing with small counts such as here.

We estimated baseline populations using a model that distinguishes between the error that affects the total size of populations and the error that affects their age distribution. Parameters for this model were obtained in a five-step procedure. In the first step, we identified the cohort of birth of the speakers who appear in each subsequent census. This was done by subtracting from the census year the upper and lower limits of the age groups for which data was collected (e.g. the speakers who appear in the age group 0–4 in 2001 belong to the birth cohort 1997–2001, and so on). Following this, we kept in our dataset the cohorts 1917–1921 through 1997–2001, i.e. those that were observed five times between 2001 and 2021. This means that counts for the speakers ages 0–4 in 2001 are obtained not only from the number of speakers observed in the age category 0–4 in the census of 2001, but also from the number of speakers observed in the age category 5–9 in the census of 2006, the age category 10–14 in the census of 2011, etc. until the age category 20–24 in 2021. The same applies for each subsequent age category in 2001 (i.e. the 5–9 based on the 10–14 in 2006, 15–19 in 2011, etc.).

In the second step, we recast in the year 2001 the counts specific to each language, cohort and census combination. This was done by adding to each count the number of speakers who potentially died between the year 2001 and the year in which a specific count was recorded. Essentially, this allowed us to have counts that were comparable in the year 2001 by being free from the influence of mortality over time. Calculations were performed using age- and period-specific probabilities of dying. Below, we explain in more detail the recast procedure (see *Recast procedure*) and describe the age- and period-specific probabilities of dying (see *Projection*).

In the third step, we estimated the total number of speakers for each language in the year 2001. This was done by summing across the cohorts 1917–1921 through 1997–2001 the counts specific to each language and census obtained in Step 2. This allowed us to obtain five separate estimates (i.e. one for each census) to which we fitted a t-distribution with location µ and scale τ (corresponding to the mean and the standard error of the five estimates available for each language), and *n*–1 degrees of freedom (where *n* is the number of estimates used, i.e. 5 in the majority of cases; see *Analyses*). Estimates for the total number of speakers specific to each census recast in the year 2001 are shown in electronic supplementary material, table S3 alongside the corresponding means and standard errors.

In Step 4, we modelled the size of each birth cohort of each language in 2001 according to negative binomial distributions (one for each cohort) with a quadratic mean–variance relationship. We chose this distribution over the Poisson one as the data showed overdispersion. Following this model, we treated speaker numbers as discrete occurrences of our event of interest and the five-year interval defining a birth cohort (e.g. 1997–2001) as the time interval in which the occurrences were to be observed. We used as inputs the mean and variance obtained from each of the five estimates available for each combination of language and cohort included between 1917–1921 and 1997–2001 obtained in Step 2. The parameters to be estimated were µ, the mean across the five censuses, and the reciprocal of the dispersion parameter $\frac{1}{\varphi }$. The dispersion parameter φ was itself defined as the variance divided by the square of the mean ($\frac{\sigma^{2}}{{µ}^{2}}$). In total, we generated 459 unique distributions, one for each cohort-language combination. Estimates for the size of each birth cohort of each language in 2001 are shown in electronic supplementary material, figure S2.

Finally, in Step 5, we rescaled the distributions by age obtained in Step 4 so that their sum matched the population size predicted by the t-distribution obtained in Step 3.

### 2.3. Trends in the mean number of child speakers per adult speaker

We derived from the baseline populations described above trends in the mean number of child speakers per adult speaker covering the period 1981–2001 and extrapolated them into the period 2006–2096. The trends for the 1981–2001 period were obtained by recasting the baseline populations into the years 1981, 1986, 1991 and 1996 and by calculating for each year the number of children that each speaker would, on average, need to raise in their language to match the numbers appearing in the data. This number thus considers simultaneously the propensity of a group of speakers to raise children in their own language and its fertility.

Like above, the recast procedure consisted of calculating the number of speakers in each birth cohort that would have been alive at each point in the past given available population and mortality estimates (see *Baseline Populations* and *Recast procedure*). The average number of children, on the other hand, was obtained by applying the *xTFR* formula proposed by Hauer and Schmertmann [45] to counts that were randomly drawn from the distributions that define the baseline populations described above. Its form is


$$xTFR=\;\left(10.65-12.55\;\pi_{25-34}\right)\times \frac{C}{W}$$

where $\pi_{25-34}$ refers to the proportion of speakers aged 25–34 among the speakers aged 15–49, *C* the number of speakers aged 0–4, and *W* the number of speakers ages 15–49. In the original *xTFR* formula, the variables $\pi_{25-34}$ and *W* refer to women instead of speakers of both genders such as here. Applying it to data referring to the entire population of a given language, we obtain the average number of child speakers per adult speaker instead of the average number of children per woman. This implies that each speaker needs to have only about one child that they will raise in their language to ensure the renewal of their language (instead of the value of 2.1 children per woman implied by the standard total fertility rate).

This formula was shown to provide accurate estimates of a population’s total fertility rate when applied to counts aggregated by five years of age at one point in time [45]. If rates of in- and outmigration and rates of language attrition are low, applying this formula to language-specific data should provide accurate estimates of the mean number of children that each adult raises in their language.

We have, following each draw from the distributions defining a language’s baseline population, five *xTFR* estimates for each language (i.e. one at each five-year interval included between 1981 and 2001). We modelled this trend by fitting one of two models. In the case of a decreasing trend (the most common one), we used a linear regression model with the logarithm of the *xTFR* value as a response variable and the year as an explanatory variable. We otherwise used a model with untransformed *xTFR* values as a response variable and the logarithm of the year as an explanatory variable. In this case, we imposed an *xTFR* value in the year 2096 that equalled the average *xTFR* value observed over the period 1981–2001 for a given language in each draw. This was done to keep the projected *xTFR* values for the period 2006–2096 within plausible boundaries. Once the model was fitted to the observed *xTFR* values, we extracted the resulting parameter values to project values at subsequent time points. In this study, we produced separate sets of results based on two different assumptions regarding future *xTFR* values. In the ‘freeze’ model, we projected *xTFR* values until the year 2046 and kept them constant thereafter. This model was meant to acknowledge the possibility that values stabilize in the future even if they have previously been decreasing or increasing. In the ‘unlimited’ model, we projected *xTFR* values until the year 2096 (the last year in which values are needed for the projection) without any constraints.

### 2.4. Recast procedure

We used a recast procedure to obtain for each language: (i) probabilistic populations in the year 2001 (i.e. baseline populations) and, for the *xTFR* estimates, (ii) estimates of the number of children speakers and childbearing age speakers in the years 1981, 1986, 1991 and 1996. (This procedure was thus applied to raw counts in (i) but to probabilistic counts in (ii)).

In the first case, let matrix $N_{c}^{t,l}$ represent the counts of speakers of language *l* in cohort *c* = {1997−2001, 1992−1996, …, 1917−1921} and census year *t* = {2006, 2011, 2016, 2021}. We obtain counts that are free from the effect of mortality by estimating the product of $N_{c}^{t,l}$ and $M_{c}^{t}$+1 where $M_{c}^{t}$ refers to the probability of people in cohort *c* dying between the year 2001 and year *t* (or, equivalently, the probability of dying between ages *2001 – c* and *t – c*). In the second case, let $N_{c}^{l}$ represent the counts of speakers of language *l* in cohort *c* = {1997−2001, 1992−1996, …, 1917−1921} obtained from a random draw from language *l*’s baseline population in the year 2001. We obtain counts in the years 1981, 1986, 1991 and 1996 by estimating the product of $N_{c}^{l}$ and $M_{c}^{t}$+1 where $M_{c}^{t}$ refers to the probability for people in cohort *c* of dying between the year *t* and the year 2001 (or, equivalently, the probability of dying between ages *t – c* and *2001 – c*). Counts in each year exclude cohorts that are yet to be born (i.e. those for which c > t).

### 2.5. Projection

Each projection run consisted of applying, iteratively to the counts of speakers by five years of age defining a language’s baseline population, the sets of age- and period-specific probabilities of dying and giving birth to a child who will eventually become a speaker of that language. Probabilities were applied within a Monte-Carlo framework to account for stochasticity. Following each iteration, speakers who die are removed from the population and those who survive are moved to the next age category (i.e. they age five years). Child speakers are added to the age category 0–4 if they survive the probability of dying specific to the first two-and-a-half years of life. The model assumes a maximum age at death of 100 years old. Each iteration covers the successive periods 2001-2006, 2006-2011, …, until the final period 2096–2101.

In any given run of the model, a language’s baseline population depends on the combination of a random sample drawn from the t-distribution (which determines a population’s size) and a series of 17 random samples (one per age group between 0–4 and 80–84) drawn from the negative binomial distributions (both were described above and determine together a population’s age structure). The age- and period-specific sets of probabilities of dying corresponded to those used for the country groups ‘Upper-middle-income countries’ (Inuktitut language) or ‘Eastern and South-Eastern Asia’ (all other languages) within the framework of the 2024 World Population Prospects (WPP) [46]. These groups were chosen as their life expectancy matched the available estimates for the Inuit population (which speaks Inuktitut) and the First Nations population (which speaks the other languages) in the year 2011 [47]. Projected probabilities for the period 2026–2101 corresponded to the WPP’s medium variant.

The age- and period-specific sets of probabilities of having a child who will be raised in the language of interest depended on the language-specific *xTFR* (mean number of child speakers per adult speaker) estimates described above. These were broken down into groups of five years of age to allow us to attribute births to specific speakers in the projection model. This breakdown resorted to sets of age- and period-specific fertility rates contained in the 2024 WPP. More specifically, we used the fertility schedules specific to all the countries and regions that had in the year 2001 a TFR of between 3 and 4 (roughly the TFR that then corresponded to the Indigenous populations of Canada [48] in that year) and fitted log-normal models to the rates across age groups in the years 2001 and 2100 (the last year of the WPP projection). We then modelled the means and standard deviations of these models as a linear function and used the predicted values at each five-year interval between 2001 and 2101 to assign births to speakers in specific age groups in our projection model.

### 2.6. Analyses

Each step in the model—including the estimation of the baseline population, the estimation of the mean number of child speakers per adult speaker, and the estimation of the longevity of each speaker—was repeated 3000 times for each language to account for the uncertainty inherent to the stochastic nature of the model and the underlying data. Analyses focused on changes over time in the number of speakers (in total and by age) and the dormancy risk of each language.

The change in the total number of speakers concentrated on the median of the values obtained across all simulation runs and the values at quantiles 0.1 and 0.9 (for the 80% projection intervals) in each year ending with 1 or 6 included between 2001 and 2101. Dormancy risk refers to the proportion of simulation runs in which the number of speakers in a chosen year is equal to zero and is expressed as a percentage. Results concentrated on the risk in the year 2101 as well as on the year in which risks reached 10 and 50% (if this happens before the year 2101). Analyses considered the risk among the whole population (i.e. the risk that the number of speakers of any ages is equal to zero), the risk among the population below 50 years old (i.e. the risk that the number of speakers aged 0−49 is equal to zero) and the risk among the population below 15 years old (i.e. the risk that the number of speakers aged 0−14 is equal to zero).

We validated our projection model by extracting the 80% projection intervals for the counts in the years 2001, 2006, 2011, 2016 and 2021, within each five-year age group, and by calculating the number of times they included the actual counts (i.e. those provided by the census) for the same years. Overall, 77% of the actual counts fell within the predicted boundaries, suggesting that our model may slightly underestimate the data’s true dispersion. The actual data fell more often within the predicted intervals in the years 2001, 2011 and 2016 (compared to 2006 and 2021) as well as in the middle age categories (compared to the younger and older ones). A complete overview is provided in electronic supplementary material, tables S4 and S5.

During any given census, some Indigenous reserves and settlements may remain incompletely enumerated because of natural hazards (e.g. wildfires) or because their inhabitants do not grant census takers permission to enter [31]. While Statistics Canada uses various methods to correct for these incomplete enumerations, estimates for some of the smaller populations (including those specific to some languages) may be less precise in some years. We compared for each language the total speaker counts (recast in the year 2001 to account for mortality) obtained from the different censuses and measured their dispersion around their mean assuming a normal distribution. Combinations of language and census years for which *p*-values were below 0.001 were removed from the estimation of the t-distributions necessary for the estimation of the baseline populations. These included the Wolastoqewi and Haida languages in the year 2001, the Ktunaxa language in the year 2006, the Mohawk language in the year 2016 and the Ktunaxa language and the Dakotan languages in the year 2021 (electronic supplementary material, table S1). Likewise, we examined how counts were distributed for each language across age groups and censuses and excluded from the estimation of the negative binomial distributions necessary for the estimation of the baseline populations the values that appeared highly unlikely. Exclusion was based on the Pearson residuals obtained by fitting chi-squared distributions separately to the counts of each language. Counts were excluded if the corresponding p-values were below 0.01 (electronic supplementary material, table S2).

All data preparation, calculations, model estimation and analyses were performed in the R environment [49]. All data and code necessary to reproduce the analyses are freely available on Zenodo [50].

## 3. Results

The 27 languages for which we present results represent less than half of the total number of Indigenous languages typically listed for Canada but include more than 97% of the whole population of Indigenous language speakers (figure 2) [51]. To ensure consistent use of the data, we modelled the speakers of certain linguistically or historically related languages together although they may now be considered distinct languages (electronic supplementary material, table S1). We estimated probabilistic baseline populations in the year 2001 and derived from them time trends in the mean number of child speakers per adult speaker (electronic supplementary material, figure 3). We then projected this trend into the future and combined it with age- and period-specific probabilities of death to estimate future population sizes in groups of five years of age at five-year intervals between 2001 and 2101 using an individual-based, cohort component projection model. Estimates for the year 2001 and the projected future values account for 3000 stochastic replications of the model (§2). We concentrate in this section on the results that we obtained based on the ‘freeze’ model used for the projection of the mean number of child speakers per adult speaker (§2.6) and refer to electronic supplementary material, tables S6 and 7 for a comparison with the results that we obtained from the ‘unlimited’ model.

### 3.1. Changes in speakers’ numbers between 2001 and 2101

In line with previous observations at the global level [52,53], baseline population sizes were highly skewed with a median of 1157 and a mean of 6021 speakers in the year 2001 (table 1). The mean number of child speaker per adult speaker of each language varied in the year 2001 from fewer than 0.01 children (Nisga’a, Wolastoqewi) to 1.9 (Atikamekw) children for each adult speaker and was in most cases projected to remain either stable or decrease over the following decades (electronic supplementary material, figure S3). Life expectancy at age 0 was 70.5 years in 2001 for the Inuktitut language speakers and 72.1 for the speakers of all other languages. Life expectancy was projected to gradually increase over time to 86.3 and 86.7 years respectively.

**Table 1.** Speaker numbers in the years 2001 and 2101. Languages are arranged in order of baseline population size; numbers between parentheses represent the values at quantiles 0.1 and 0.9 in the probabilistic baseline populations and the projections, respectively.

language name	year 2001	year 2101
Haida	73 (54−100)	0 (0−7)
Tlingit	92 (77−110)	3 (0−56)
Ktunaxa	101 (95−108)	0 (0−16)
Tsimshian	252 (200−320)	0 (0−22)
Gwich'in	306 (258−363)	3 (0−32)
Nuu-chah-nulth	312 (256−383)	9 (0−38)
Mohawk	321 (282−364)	52 (5−237)
Ntlakapamux	362 (292−448)	3 (0−25)
Wolastoqewi	442 (346−573)	0 (0−1)
Nisga'a	506 (437−591)	0 (0−0)
Secwepemctsin	540 (415−708)	63 (19−155)
Tsilhqot'in	767 (639−908)	18 (5−63)
Gitxsan	889 (764−1,044)	9 (1–32)
Dakelh	1,157 (934–1,437)	26 (7−76)
Anicinabemowin	1,307 (1,087–1,578)	277 (93−1,079)
Tlicho	1,717 (1,552–1,912)	103 (64−173)
Slavey (N & S)	2,147 (1,883–2,436)	70 (37−139)
Blackfoot	2,875 (2,711–3,048)	59 (20−160)
Dakotan langs	4,125 (3,637–4,682)	1,288 (437–5,086)
Atikamekw	4,230 (3,892–4,588)	9,276 (,5314–18,042)
Mi'kmaq	6,181 (5,396–7,129)	2,824 (1,666–5,239)
Innu-Naskapi	8,690 (7,688–9,793)	11,678 (7,150–20,426)
Dene	8,692 (7,736–9,712)	7,478 (4,551–13,663)
Oji-Cree	8,987 (8,056–10,074)	3,782 (2,668–5,774)
Ojibwa langs	17,954 (15,110–21,506)	1,130 (744–1,785)
Inuktitut	25,846 (23,546–28,210)	59,811 (40,182–92,486)
Cree langs	63,708 (55,769-73,139)	27,648 (18,623-42,904)
**Total**	**1 63 190 (153 568−174 662**)	**1 33 915 (106 939–171 929**)

**Figure 3.**
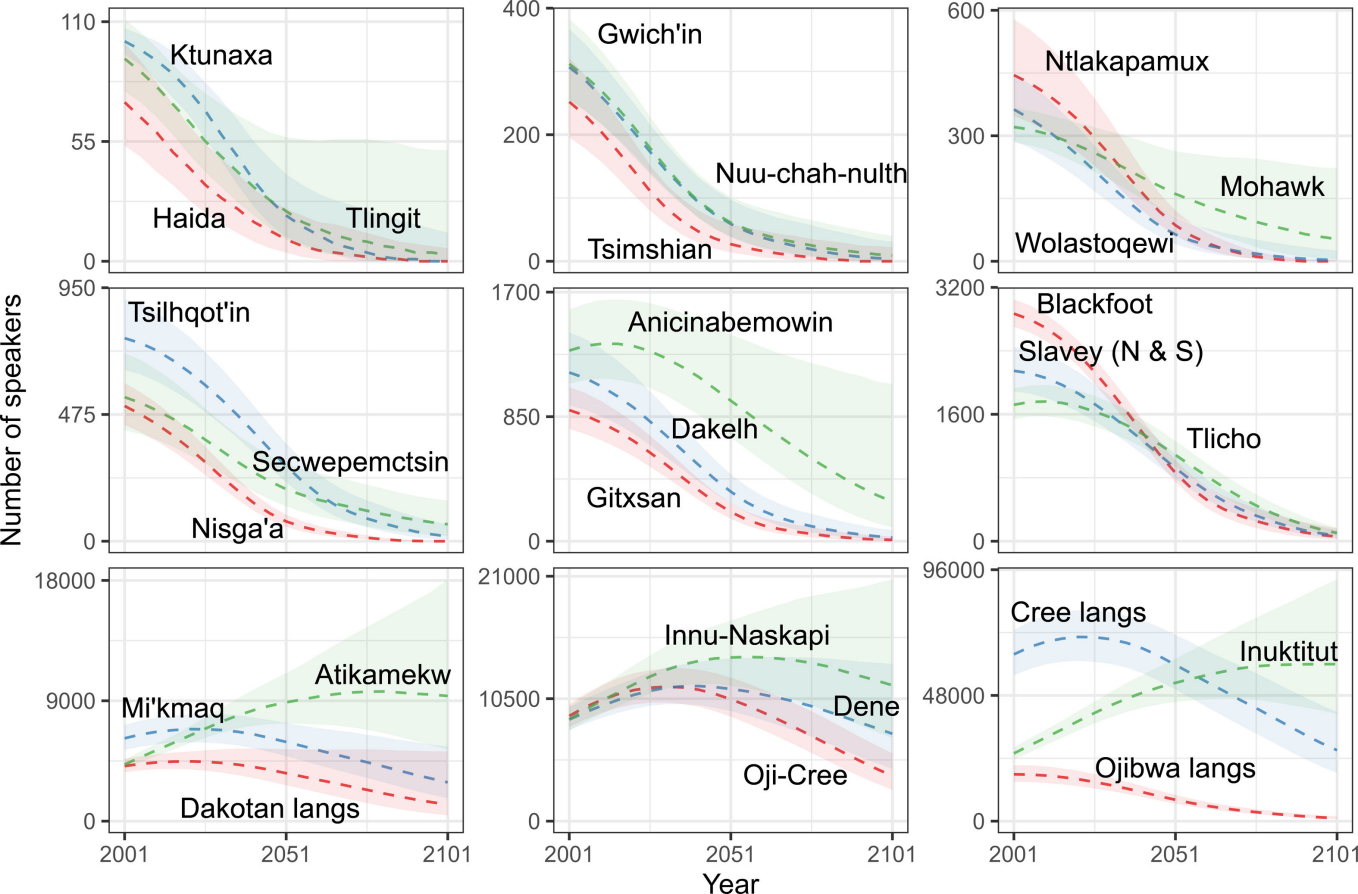
Total number of speakers for the period 2001−2101. Each dotted line represents the median of the projected values across the 3000 runs, the shaded areas the 80% projection intervals. Languages are grouped by baseline population size and groups are arranged in order of increasing baseline population size (from top left to bottom right).

Applying these trends to the baseline populations, we find decreases in total speaker numbers in most languages, including in all the 18 languages with fewer than 3000 speakers at baseline (figure 3). Total speaker numbers are projected to increase in only three languages (Atikamekw, Innu-Naskapi, Inuktitut) over the period of interest, which is the result of them currently having younger age structures and higher projected mean numbers of child speakers per adult speaker. Another small group of languages are projected to initially gain speakers to then return to sizes that are slightly or much lower than their initial population sizes (Anicinabemowin, Dene, Mi’kmaq, Oji-Cree, Tlicho and the Cree and Dakotan languages).

Among the 18 languages projected to follow a strictly descending trajectory, all except two (Mohawk and Secwepemctsin) are projected to lose more than 90% of their population during the 2001−2101 period (table 1). Among these, five are projected to lose all their speakers, with medians in the year 2101 reaching a value of zero for Haida, Ktunaxa, Tsimshian, Wolastoqewi and Nisga’a. Eighty percent projection intervals include zero speakers in four other languages (Tlingit, Gwich’in, Nuu-chah-nulth, Ntlakapamux). Some languages with initially larger populations will lose large shares of their initial population without being directly at risk of losing all their speakers: Tsilhqot’in is projected to have 18 (PI 5−63) in the year 2101, Gitxsan 9 (PI 1−32), Dakelh 26 (PI 7−76), Slavey 70 (PI 37−139) and Blackfoot 59 (PI 20−160). Conversely, the Atikamekw and Inuktitut languages, which are projected to follow an ascending trajectory, could see their population more than double during the projection period. These increases, combined with the relatively stable pathways in some of the other larger languages (e.g. Cree languages, Oji-Cree, Innu-Naskapi, Dene, Mi’kmaq, Dakotan languages) are contributing to maintaining the total size of the population of Indigenous language speakers of Canada roughly constant during the projection period.

### 3.2. Dormancy risks in the year 2101

We estimated a language’s dormancy risk as the percentage of times (out of 3000) it is projected to have zero speakers in each year (table 2). Considering the speakers of all ages, we found a total of 14 languages with non-zero dormancy risk in the year 2101, including risks above 50% for Nisga’a (95.2%), Wolastoqewi (82.6%), Haida (74.4%), Tsimshian (65.0%) and Ktunaxa (61.6%). Risks start increasing relatively late in the projection period, however, with the 10% threshold being passed the first time in the year 2066 (Haida). Meanwhile, 19 languages have a non-zero risk of having no speakers below age 50 in the year 2101 and 20 have a non-zero risk of having no speakers below age 15. For Nisga’a, Wolastoqewi, Haida, Tsimshian and Ktunaxa speakers, the window for ensuring that children continue to learn the language (i.e. the period during which there are still speakers below age 15) has likely already closed while it is about to close in a handful of other languages including Gwich’in, Tlingit, Ntlakapamux, Nuu-chah-nulth and Gitxsan. The window for reestablishing the intergenerational transmission of the same languages, assuming it becomes interrupted (i.e. the period during which there will be speakers below age 50), will start closing around the middle of the current century. Other languages (e.g. Tsilhqot'in, Dakelh, Tlicho, Slavey, Blackfoot) face low risks of having lost all their speakers by the year 2101 but could still lose them at some point in the beginning of the 22nd century as they are projected to swiftly progress towards higher risks of having lost their population below 50 and 15 years old.

**Table 2.** Dormancy risk in the year 2101. Numbers indicate the percentage of simulation iterations (out of 3000) in which the number of speakers in the age range indicated at the top of each column is equal to zero in the year 2101. Numbers between parentheses indicate the years in which the 10 and 50% values are reached. (For example, 95.2% of the simulations ran for Nisga’a indicated no speakers left in the year 2101; the 10% value was reached in the year 2086, the 50% one in the year 2096).

language name	risk of no speakers of any ages	risk of no speakers below age 50	risk of no speakers below age 15
Nisga'a	95.2 (2086−2096)	99.9 (2046−2046)	99.9 (2011−2011)
Wolastoqewi	82.6 (2086−2091)	96.5 (2041−2046)	98.5 (2006−2011)
Haida	74.4 (2066−2091)	84.7 (2031−2046)	90.4 (2001−2011)
Tsimshian	65 (2081−2091)	77.5 (2041−2046)	85.3 (2006−2011)
Ktunaxa	61.6 (2076−2096)	76.7 (2036−2051)	85.9 (2001−2016)
Gwich'in	33.5 (2091-)	57.7 (2046−2086)	76.6 (2011−2046)
Tlingit	32.8 (2081-)	51 (2041−2101)	66.6 (2006−2051)
Ntlakapamux	28.1 (2091-)	61.1 (2051−2086)	82.6 (2016−2046)
Nuu-chah-nulth	11.8 (2101-)	37.5 (2061-)	65.2 (2021−2076)
Gitxsan	6.9 (-)	56.5 (2066−2096)	86.3 (2031−2056)
Mohawk	1.7 (-)	22.7 (2081-)	37 (2046-)
Tsilhqot'in	0.3 (-)	49.5 (2076-)	80.2 (2041−2066)
Dakelh	0.2 (-)	36.5 (2081-)	68.2 (2046−2081)
Secwepemctsin	0.1 (-)	4.8 (-)	17.7 (2081-)
Tlicho	0	24 (2096-)	88.6 (2061−2076)
Slavey (N & S)	0	26.7 (2091-)	81.5 (2056−2081)
Blackfoot	0	25 (2091-)	62.2 (2051−2091)
Anicinabemowin	0	1.8 (-)	19.9 (2086-)
Dakotan langs	0	0.2 (-)	6.7 (-)
Ojibwa langs	0	0	2.2 (-)

## 4. Discussion

**Figure 4.**
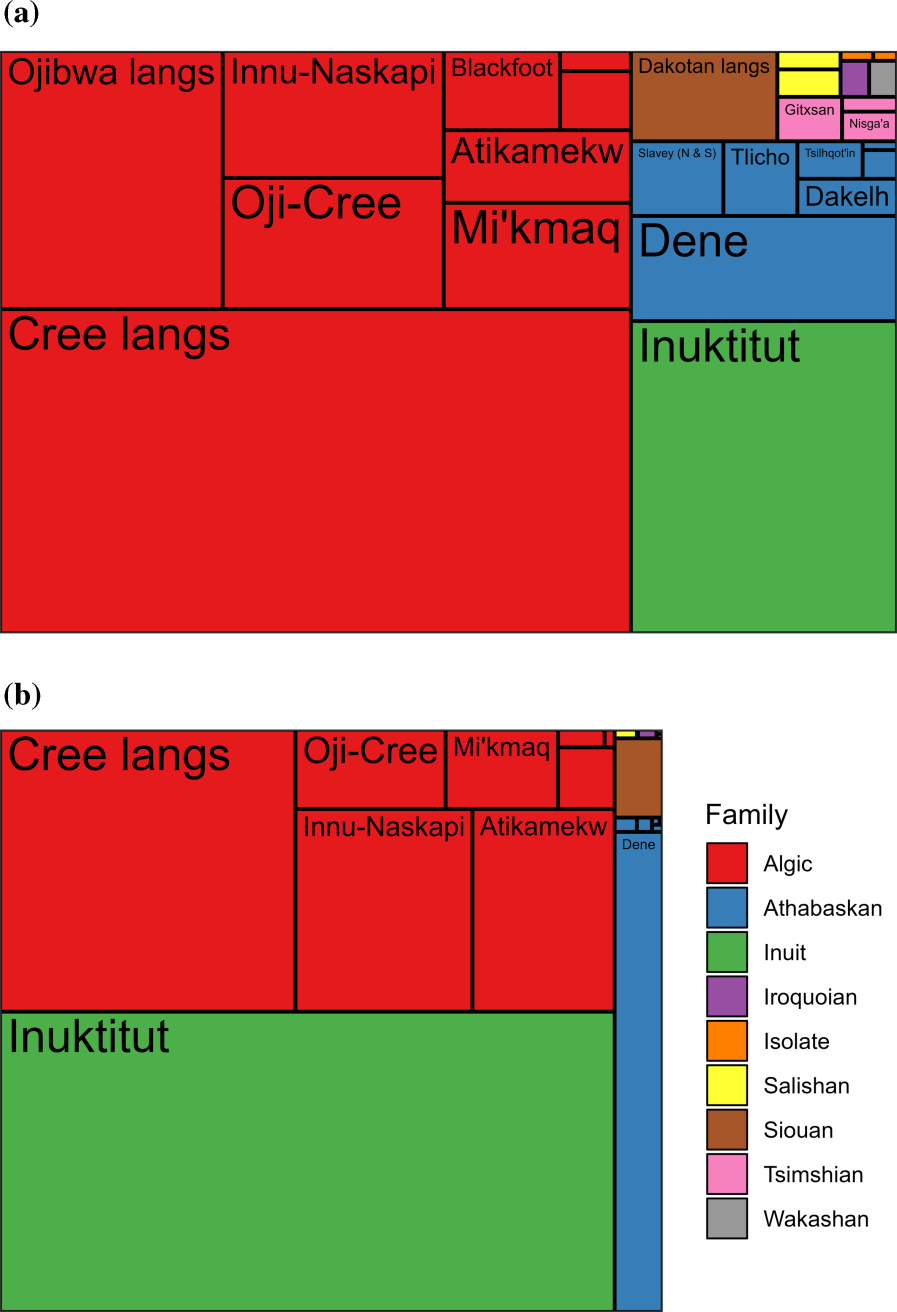
Total population sizes in the year 2001 (a) and 2101 (b; estimated and projected medians) for each language, by family. The sizes of the rectangles in both panels are proportional to the total population sizes in the corresponding years.

Our demographic projection model suggests drastic decreases in the number of speakers of most of the 27 Indigenous languages considered here between the years 2001 and 2101. For many languages, there are considerable risks of slipping into dormancy if the current trends in the way that speakers have been transmitting their language to their children persist into the future. These decreases concern mostly the languages that already had small populations at the start of the projection period. Conversely, most languages that had larger populations in the year 2001 are projected to either gain speakers or lose them at a much slower pace. As a result, it is likely that speakers will be distributed much differently across the different Indigenous languages of Canada at the end of the current century compared to now (figure 4).

For example, in 2001, the nine largest languages accounted for 91.5% of the entire population of Indigenous language speakers considered here; in 2101, they could account for 99.6%, in a median scenario. Among these nine languages, some might see their relative importance grow while others might see it decrease. For example, Inuktitut is projected to surpass the Cree languages as the one with the largest number of speakers while the Atikamekw and Innu-Naskapi languages could surpass the Oji-Cree and Ojibwa languages as the languages with the third and fourth largest numbers of speakers. This possible reconfiguration of the way that speakers are distributed across languages could also impact the size of language families. The Algic and Inuit families should continue to be the most spoken ones, but all the other families could see their size strongly diminish with some even risking disappearing entirely. For example, the total number of speakers found across the three Tsimshian languages could be as small as eight in a median scenario in the year 2101. Meanwhile, the isolates Haida and Ktunaxa are projected to both have zero speakers in a median scenario in the year 2101 and are among the languages that are the most at risk of dormancy.

Not only are reductions in speaker numbers more highly concentrated among certain language groups, but there also appears to be a strong geographical patterning in endangerment. We note for example generally higher dormancy risks among the languages originally spoken in the western part of Canada than among those spoken in the eastern part. This could likely be the result of the country’s specific colonization history combined with longer-term language diversification dynamics [54]. At any rate, our results echo previous studies by identifying more widespread dormancy risks among isolated languages or languages that are phylogenetically or geographically close [52,55–57]. These unequal patterns of language loss not only have important implications for the affected communities, but also for science, as losing isolates, entire language families and geographic regions may mean losing languages with unique characteristics, which in turn means the loss of opportunities for obtaining a better understanding of human cognition and history.

Previous studies have explored the future of linguistic diversity based on Ethnologue’s classification by endangerment status [17,18], which itself rests on the Expanded Graded Intergenerational Disruption Scale (EGIDS) [16]. For example, one study estimated the future rate of language dormancy by assuming that languages in the EGIDS category 8b (stating that the youngest speakers are elderly) would all be dormant by the year 2033, those in the category 8 a (the youngest speakers are grandparents) would be so by the year 2058, and those in the category 7 (the youngest speakers are of the childbearing generation) would be dormant by the year 2083 [18]. In contrast, by using census data and demographic projection, we were able to account for the number of speakers and age structure specific to each language, which in turn allowed us to provide more precise potential dormancy years. Using this approach, we were also able to provide estimates for languages that were still being transmitted to the younger generation (i.e., languages below EGIDS 7) at the turn of the last century. Importantly, our approach provided projection ranges for the number of speakers that languages might have in the future, something that has to our knowledge not been done concerning Indigenous or endangered languages. Also, the explicit consideration of time in our model, including the consideration of both the age of the speakers and calendar time, provides a detailed timeframe for intervention.

Our approach, however, possesses limitations that should be borne in mind. The results of our projection model are bound by the assumptions found at its base. An important assumption regarding the results presented here concern the continuation and stabilization from 2046 onwards of the language-specific trends in the mean number of child speakers per adult speaker. Additional results shown in electronic supplementary material, tables S6 and S7 suggest that the choice of ‘freezing’ the trends captured by our model—over letting them evolve unchecked—has little bearing on the results and mainly affects larger languages. This result underscores the importance of the way that speakers are currently distributed across age groups for determining how their language will fare in the future. However, all results presented here reflect a certain continuation of the past trends in the propensity of speakers to raise children in their own language. Yet, it cannot be excluded that these trends may accelerate in the future and thus speed up the rate of language loss. Alternatively, they could reverse if language preservation efforts are successful and translate into a slower rate of language loss. Either way, any major changes in the way that speakers have been transmitting their language to their children could translate into an important departure from the numbers that we have provided here. Therefore, all our results should be seen as an indication of what could happen under the assumption of no major changes in historical trends.

Another potential caveat concerns the validity of the data on which we based our projection. The census data we used here captures what speakers consider to be their first language, but such self-assessments may have different meanings for different speakers or can be interpreted differently among different data users. As a result, there is a possibility that our results would differ if we were to use data that was collected with different survey methods. Furthermore, our estimates rest on the assumption that speakers retain the capacity to use their first language throughout their life but language attrition may accelerate the process of language dormancy if speakers are too few to have frequent opportunities to use their language [58].

Importantly, estimates of speaker numbers and dormancy risks are only one criterion against which revitalization efforts may be evaluated. Language revitalization is a holistic process aimed at improving a community’s overall well-being, rather than merely increasing speaker numbers [59]. Evaluating a language’s vitality should consider the context in which the language is spoken and the needs and wishes of each community [60]. As such, our estimates should not be used on their own but as a complement to local knowledge. Furthermore, due to data unavailability, our analyses considered less than half of all Indigenous language varieties of Canada, meaning that we are not in a position to assess dormancy risks in several languages.

Nonetheless, we consider that our study shows the potential usefulness of demographic projection for evaluating the current vitality of languages. One key requirement is counts of speakers by age, but relatively few countries disseminate such data systematically accounting for their population’s entire diversity, including in terms of its Indigenous and minority languages. We thus encourage more statistical agencies around the world to expand the dissemination of high-quality linguistic data in a way that properly accounts for the whole of the planet’s linguistic diversity. By doing so, we might be able to better pinpoint where and how fast we are losing our rich cultural heritage.

## Data Availability

Data and code are available at Boissonneault M. Projected speaker numbers and dormancy risks of Canada's Indigenous languages. Zenodo; [61]. Supplementary material is available online [62].

## Footnotes

^1^
Throughout this paper, language names correspond to those used by Statistics Canada following the census of 2021. Since we are aware that these names do not necessarily have consistent usage across various contexts, we refer to the ISO code correspondences provided in electronic supplementary material, table S1 in case of ambiguity.
